# Neurosurgical management of non-spastic movement disorders

**DOI:** 10.1007/s00381-023-06100-1

**Published:** 2023-07-31

**Authors:** Sean D. McEvoy, David D. Limbrick, Jeffrey Steven Raskin

**Affiliations:** 1 Department of Neurological Surgery, Washington University School of Medicine in St. Louis, Brookings, MO USA; 2 Department of Neurological Surgery, Northwestern University Feinberg School of Medicine, Chicago, IL USA; 3 Division of Pediatric Neurosurgery, Ann & Robert H. Lurie Children’s Hospital, Chicago, IL USA

**Keywords:** Deep brain stimulation, Movement disorders, Pallidotomy, Rhizotomy

## Abstract

**Background:**

Non-spastic movement disorders in children are common, although true epidemiologic data is difficult to ascertain. Children are more likely than adults to have hyperkinetic movement disorders defined as tics, dystonia, chorea/athetosis, or tremor. These conditions manifest from acquired or heredodegenerative etiologies and often severely limit function despite medical and surgical management paradigms. Neurosurgical management for these conditions is highlighted.

**Methods:**

We performed a focused review of the literature by searching PubMed on 16 May 2023 using key terms related to our review. No temporal filter was applied, but only English articles were considered. We searched for the terms ((“Pallidotomy”[Mesh]) OR “Rhizotomy”[Mesh]) OR “Deep Brain Stimulation”[Mesh], dystonia, children, adolescent, pediatric, globus pallidus, in combination. All articles were reviewed for inclusion in the final reference list.

**Results:**

Our search terms returned 37 articles from 2004 to 2023. Articles covering deep brain stimulation were the most common (*n* = 34) followed by pallidotomy (*n* = 3); there were no articles on rhizotomy.

**Discussion:**

Non-spastic movement disorders are common in children and difficult to treat. Most of these patients are referred to neurosurgery for the management of dystonia, with modern neurosurgical management including pallidotomy, rhizotomy, and deep brain stimulation. Historically, pallidotomy has been effective and may still be preferred in subpopulations presenting either in status dystonicus or with high risk for hardware complications. Superiority of DBS over pallidotomy for secondary dystonia has not been determined. Rhizotomy is an underutilized surgical tool and more study characterizing efficacy and risk profile is indicated.

## Introduction

Movement disorders are defined as involuntary motor activity and are phenomenologically determined hyperkinetic, hypokinetic, and ataxic [1, 2]. Pediatric movement disorders (PMDs) manifest in patients less than 18 years of age and are most commonly hyperkinetic and, excluding spasticity, are subcategorized as chorea/athetosis, dystonia, myoclonus, tics, and tremor [1–3]. The pathophysiology of spasticity is understood to be a constitutively active series of myotatic reflexes which is generally associated with decreased segmental corticospinal input as a result of either brain or spinal pathology; however, the pathophysiology for non-spasticity associated hypertonia syndromes manifests from the brain only and involves dysregulation of the many subcortical nuclei contributing to movement selection [2].

Although specific epidemiologic data is lacking, PMDs are among the most common reason for presentation to a pediatric neurology clinic [4]. Tics are the most common manifestation followed by dystonia [5]. Tics, stereotypies, and psychogenic PMDs generally are treated non-surgically as most, and specifically tics, improve with age [6]. When the PMD is acquired due to a precipitating etiology, the appropriate management targets the underlying condition first; examples include immunologic and infectious conditions [2, 4].

The genetics underlying heredodegenerative PMDs is ever expanding. Primary dystonia is defined by an autosomal dominant chromosome 9 GAG deletion in the DYT-1 gene, has an incidence of 16–117 patients per million and was first identified in 1989, and since then, more than 20 “DYT” genes have been identified [7–9]. While primary dystonia is known to be associated with significant benefit from both pallidotomy and DBS, the response from other DYT allotypes remains to be completely characterized and is complicated by inconsistent naming and a lack of causative pathology [10]. Technological advances in genotyping have precipitated a rapid expansion in the identification of abnormal alleles (i.e., Glut-1), with incomplete understanding of the associated causation of pathological movements or effective therapeutic strategies. Secondary dystonia caused by an underlying etiology is manifest from a heterogeneous group of conditions including cerebral palsy, anoxia/hypoxia, central nervous system (CNS) infections, infarction, myoclonus dystonia, tardive dystonia, pantothenate kinase deficiency, metabolic diseases, mitochondrial diseases, Leigh syndrome, and trauma [11, 12]. Idiopathic dystonia refers to forms of dystonia which have no identifiable cause and most likely have undiagnosed genetic underpinnings [13]. 

PMDs lack discrete biomarkers, therefore characterization relies on clinical evaluation and grading systems. The final common pathway for hyperkinetic conditions is hyperactivation of striated muscle and rigidity, and this can be dynamic often manifesting in a twisting of extremities caused by activation of agonist and antagonist muscles [2]. Chronically activated muscles cause muscle breakdown, fibrosis, bone remodeling, and joint dysfunction. Structural musculoskeletal abnormalities called contractures can limit function, cause pain, and impact sitting position and brace efficacy. Kyphoscoliosis can progress quickly to limit cardiopulmonary function while chronic hip dislocation causes significant pain and can precipitate worse hypertonic crises.

**Fig. 1:**
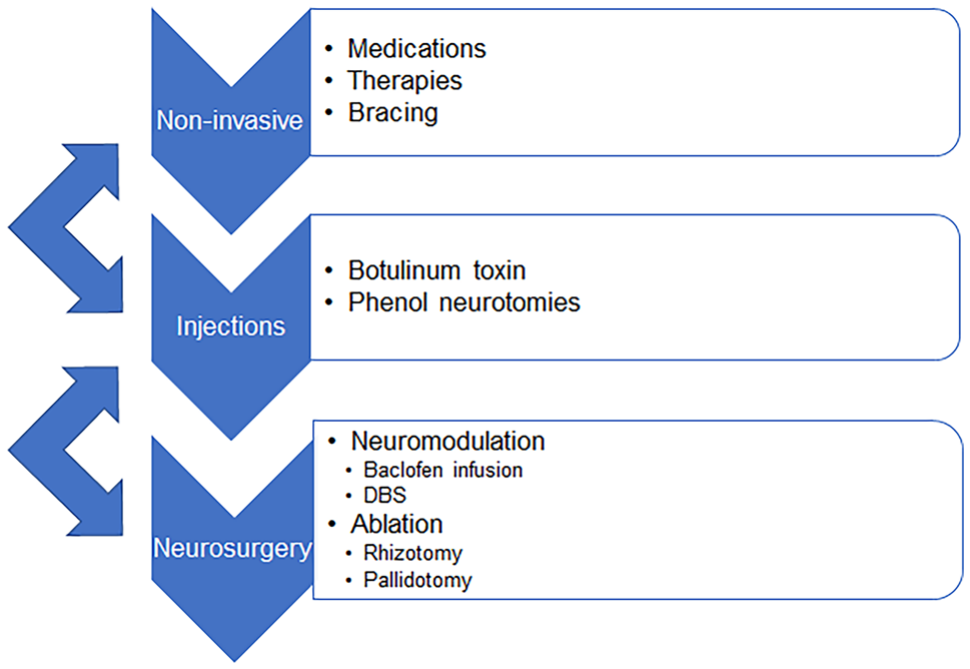
Iterative and reciprocal algorithm for the medico-surgical management of hypertonia in children

Diagnosis of PMDs occurs across specialties with patients managed by clinicians in primary care, physiatry, neurology, and neurosurgery. The treatment algorithm generally includes enteric antispasmodic and centrally acting medications, bracing and splinting, therapies, injections with botulinum toxin, or phenol nerve crenation [14–17]. Patients who are medically refractory, rapidly progressive or have a primary dystonia may be referred to a multi-disciplinary movement disorder clinic for consensus management, described in Fig. 1.

The genesis of modern stereotactic neurosurgery in humans is based on intentional lesioning of basal ganglia circuitry to affect movement disorders in adults with Parkinson’s disease (PD) [18]. The evolution of the stereotactic frame paired with early neuroimaging in the form of air, and then subsequently contrast ventriculography, allowed for reproducible targeting of subcortical nuclei and modification of the extrapyramidal motor system [19]. Drs. Benabid and DeLong are credited for their award-winning work in understanding the intricacies of normal and pathophysiological connectivity of the basal ganglia circuitry, the canonical mechanism we use today to understand the neural correlates to movement disorders [20]. From the 1930s to the 1950s, thalamotomy and pallidotomy were primarily performed in adult patients with PD and tremor, experiencing a sharp decline as Levodopa came to market [21, 22]. Thalamotomy was replaced by more effective pallidotomy, and evolution of surgical techniques advanced from chemopallidectomy to the use of physiological feedback using radiofrequency ablation (RFA) [23]. 

During the era of RFA for the treatment of movement disorders (predominately in the 1960s), surgeons often performed electrical stimulation of basal ganglia structures concurrently with ablation procedure [24]. The changes in movement resulting from the stimulation assisted with confirmation of the electrode location prior to performing permanent lesioning. This early experience with brain modulation from stimulation was performed either in the operating room or temporarily through leads tunneled to an external generator. This practice helped to refine location and characteristics of stimulation parameters to help maximize response [25]. 

The development of implantable cardiac pacemakers helped to facilitate the technological innovation of a permanently implantable generator for brain stimulation [26]. The FDA first approved a deep brain stimulation (DBS) device in 1997 to target the ventralis intermediate nucleus (Vim) of the thalamus to treat essential tremor. This was followed by further approval in 2002 for treatment of the symptoms of Parkinson’s disease through subcortical stimulation of the subthalamic nucleus (STN) or globus pallidus internus (GPi). The first reported case of DBS in a child occurred in 1996, and after much further investigation in 2003, the FDA further expanded the approval of DBS to dystonia including children 7 years of age and above under a humanitarian device exemption (HDE) [27]. 

Herein, we explore several neurosurgical tone reduction paradigms for medically refractory non-spastic hypertonia which include pallidotomy, rhizotomy, and deep brain stimulation.

### Neuroablative therapies for pediatric movement disorders

The modern medical management algorithm in Fig. 1 is commonly effective for hypertonia; however, the PMD inevitably progresses or is worsened by physiologic growth which causes musculoskeletal dysfunction, physical disability, poor quality of life, and premature death [5, 28–30]. Neuroablative therapies for PMDs include CNS lesions and rhizotomy.

### Primary and secondary dystonia

Generalized dystonia remains a difficult entity to treat, in part due to the heterogeneity of the underlying diagnoses [30]. The application of pallidotomy and thalamotomy in children is uncommon and the vast majority of applications is in patients with generalized primary or secondary dystonia, with the first case series published in 1976 [31]. The youngest patient treated with bilateral pallidotomy for GLUT-1 deficiency was 18 months of age and had initial benefit in hypertonia symptoms [32]. A recent review of the literature evaluating 40 generalized secondary dystonic patients receiving unilateral or bilateral pallidotomy only included four children in 40 patients [33]. Case series featuring both adult and pediatric patients do not show an age-dependent difference in outcomes [33]. 

Pooled level III evidence suggests that patients with primary generalized dystonia respond much better than those with secondary dystonia [34]. Secondary dystonic patients remain recalcitrant to medico-surgical techniques. Unfortunately, they respond significantly less to thalamotomy or pallidotomy than primary dystonic patients [5, 23]. A modern series of 10 children with mixed etiologies for secondary generalized dystonia treated at a single center showed a moderate efficacy and safety profile. No patients had worsening symptoms following pallidotomy; while three died following intervention they were from COVID-19 and Wilson’s disease-related liver failure, with only one death 2 years following pallidotomy-related bulbar dysfunction. The Burke‐Fahn‐Marsden dystonia rating scale (BFMDRS) was reduced 25.5% on average 1 year following the procedure with some responders achieving 40% reduction of dystonia [5]. 

Features of dystonia which are positively correlated with improvement following pallidotomy include a primary neurogenetic condition, MRI “lesion-negative,” and shorter course of disease [34]. Bulbar dysfunction is a known consequence of bilateral pallidotomy, and this morbidity profile is considered better than that from thalamotomy [5, 23]. The reduction of dystonia in the pediatric population is modest based on the handful of case series that exist in the literature [34–38]; the small number of case series in context of the many decades of available therapy may highlight a publication bias against negative results or poor outcomes. The lack of standard grading scales in this population makes general conclusions difficult regarding efficacy. Complications are overall low with bulbar dysfunction being the most severe, with overall lower morbidity compared with thalamotomy [23]. 

### Mixed dystonia and hyperkinetic conditions

The majority of the literature for thalamotomy and pallidotomy is for management of refractory dystonia in children. The lack of common biomarkers requires PMDs to be diagnosed phenomenologically, and dystonia is a common and easily identifiable clinical feature. It is also increasingly genetically defined [10]; however, combinations of dystonia and chorea, ataxia, hemiballismus, myoclonus, and tremor are often present. In many of these cases, linkage analysis identifying the chromosomal location with a modified naming convention is being studied to facilitate more clinically relevant grouping [10, 39]. For example, chorea is a movement disorder that causes sudden, unintended, and uncontrollable jerky movements of the arms, legs, and facial muscles. Chorea is usually a symptom of Huntington’s disease (HD), but can be induced in rheumatic fever complications, known as Sydenham’s chorea, by other disorders, certain medicines (including levodopa or some antiseizure or antipsychotic drugs), metabolic and endocrine disorders such as hyperthyroidism, or vascular diseases [40]. The first pallidotomy may have in fact been done in 1948 to ameliorate choreiform movements in HD, specifically [41]. For patients with neurodegenerative conditions like Wilson’s disease and HD, ablative therapies may be initially therapeutic but fail in the long term due to the progressive nature of the disease. Similarly, pallidotomy for the management of segmental or focal limb hypertonia in children is lacking.

### Modern pallidotomy procedural steps

Modern pallidotomy is performed using multiple platforms. Avascular trajectories are planned on a stereotactic imaging platform; images include T1- and T2-weighted MRI sequences, as well as T1 post-gadolinium and sometimes a high contrast T2 sequence (e.g., FGATIR). Images are carefully merged with trajectories either directly targeting the GPi or modified based on typical AC-PC coordinates. Asleep robotic or frame-based stereotaxy is performed to place the RFA needle or laser fiber to target and microelectrode recording is uncommon [42–44]. RFA ablation parameters vary somewhat but usually include multiple co-axial lesions. Subcortical targeting for pallidotomy is historically and most commonly reported in the posteroventral pallidum, although targeting of the midportion of the GPi to maintain the posteroventral GPi for future DBS has been reported to be effective in status dystonicus [45]. 

### Rhizotomy

While pallidotomy can be effective for generalized hypertonia, its application in segmental or limb-specific hypertonia is lacking and may be addressed by more peripheral lesioning. For example, torticollis in children has many etiologies with trauma and post-pharyngitis infection being the most common. In these cases, management includes treating the underlying infection as indicated, and immobilizing with a cervical orthosis often with antispasmodic medication like benzodiazepine. When it manifests because of cerebral palsy or focal dystonia, it may be treated by peripheral cervical rhizotomy with good effect [46]. 

Even in the presence of generalized mixed hypertonia, patients may choose to be managed with rhizotomy targeting cervical and lumbosacral denervation. Cervical rhizotomy for spasticity was performed as a logical extension during the expansion of indications for rhizotomy in the surgical management of spasticity during the 1970s–1980s. In a small series of rhizotomy reported by Laitinen [47], one of eight underwent cervical dorsal rhizotomy where C6–8 dorsal roots were partially sectioned without ventral root sectioning leading to minimal benefit; a larger series of 13 adult patients and 15 upper extremities underwent brachial plexus dorsal rhizotomy and showed improved function and tone without hand anesthesia [48]. A review on the neurosurgical management of spastic conditions of the upper extremity suggests that partial or palliative dorsal rhizotomy (without selectivity) can be an effective therapy for tone control but is not a reliable treatment to improve function [49]. 

In the setting of mixed hypertonia, it is necessary to add ventral root sectioning [50]. The optimal extent of ventral root sectioning remains unknown, and this surgical approach is uncommonly performed. Non-ambulatory and total care patients often have a medically refractory mixed hypertonia, and in the modern era these patients are often treated with intrathecal baclofen (ITB) therapy [51]. ITB is not a panacea; some families decline ITB, some children are not candidates, and some PMDs remain refractory to ITB. In these relatively few cases, a ventral–dorsal rhizotomy may be beneficial [50]. 

The first cervical rhizotomies for mixed hypertonia pre-date ITB therapy. In the 1970s, dorsal cervical rhizotomies were found to be effective for dystonia, athetosis, and spasticity [52, 53]. Short-term results showed decreased hypertonia in most cases, with subsequent larger case series demonstrating less efficacy for athetosis following dorsal C1–5 rhizotomy [54]. Ventral rhizotomy in adults performed for torticollis was reported to have fewer sensory deficits than the Bertrand procedure [55]. Albright and colleagues presented a series of six children who uniformly benefitted from both cervical and lumbosacral approaches to limb denervation via ventral–dorsal rhizotomy [50]. 

There is a paucity of clinical research investigating cervical and lumbosacral ventral–dorsal rhizotomy regarding the long-term effects on PMDs, complications, cost and hospital resource utilization, association with need for further medico-surgical management, and patient satisfaction dimensions. Because of these knowledge limitations, ventral–dorsal rhizotomy remains an option for, and is not a preferred method of management for, dystonia. Practitioners should be mindful that ventral–dorsal rhizotomy treats dystonia at a common final pathway of all hypertonia, by decreasing peripheral nerve activation, and it does not address the root cause of dystonia which is due to dysregulated extrapyramidal pathophysiology. The authors’ own experience with this condition is overwhelmingly positive, with notable, yet unpublished, very positive reported patient and family satisfaction. Due to many unknown clinical factors for ventral–dorsal rhizotomy, it may be beneficial to obtain an ethics consultation prior to performing this irreversible surgery.

### Ventral–dorsal rhizotomy procedural steps

Cervical and lumbosacral ventral–dorsal rhizotomy can be performed in the same surgical setting if indicated, or separately. In both cases, the patient is carefully positioned prone with subdermal electrodes in the appropriate muscle groups, followed by antibiosis, sterile preparation and draping to include the incision(s), and a team pause. The cervical approach is performed via bilateral paraspinal muscle dissection, osteoplastic laminotomy, and dural opening. We leave the dorsal bony elements attached at the cephalad interspinous and supraspinous ligaments; although, they can be primarily explanted and reattached. Recall that the cervical mixed nerve roots exit above the correspondingly numbered vertebral body, so we perform a C5 to T1 osteoplastic laminoplasty to access the C6, C7, C8, and T1 mixed nerve roots. We spare C3–5 due to their uncommon involvement in the movement disorder and the overlapping phrenic nerve contribution. Intradural microneurosurgical visualization is used to identify candidate nerves. Beginning on one side, we use Gilette nerve hooks to stimulate C6 and threshold both the dorsal and ventral roots, correlating to the intended myotome. We then section 80–90% of the dorsal and ventral roots using visual estimation. Minor bleeding is controlled with gel foam. We often cut the denticulate ligaments to facilitate mobilization of the spinal cord and improved access to the ventral roots. This is performed for C6–T1 bilaterally. Upon completion, we close the dura in standard water-tight fashion, obtain hemostasis, and perform an osteoplastic laminoplasty using a proprietary system or sutures depending on bone quantity/quality. The soft tissue is closed in typical layered fashion.

The lumbosacral approach is similar to the Park approach for SDR [56], and our incision is placed over T12–L1. We perform a similar paraspinal muscle dissection and osteoplastic laminotomy and dural opening. We place a blue background behind the conus and available cauda equina. Beginning at L1, we threshold across the mixed nerve and then each ventral and dorsal root to confirm the associated myotome. We then section 80–90% of each root at L1 and then place the remaining portion behind the background. We then perform the same microneurosurgical dissection for the dorsal roots from L2 to S1, threshold them, and then section 80–90% and place the remaining portion behind the background. Following dorsal sectioning, we identify the ventral roots in similar fashion, section 80–90%, and place them behind the background. We avoid sectioning S2–5 or any nerve with strong bilateral sphincteric activation. The same procedure is performed on the contralateral side. We evaluate the filum terminale and if it does not stimulate and is easily accessible, we section the filum and send it to pathology for evaluation. We then close in typical fashion as previously stated.

### Example case

**Fig. 2:**
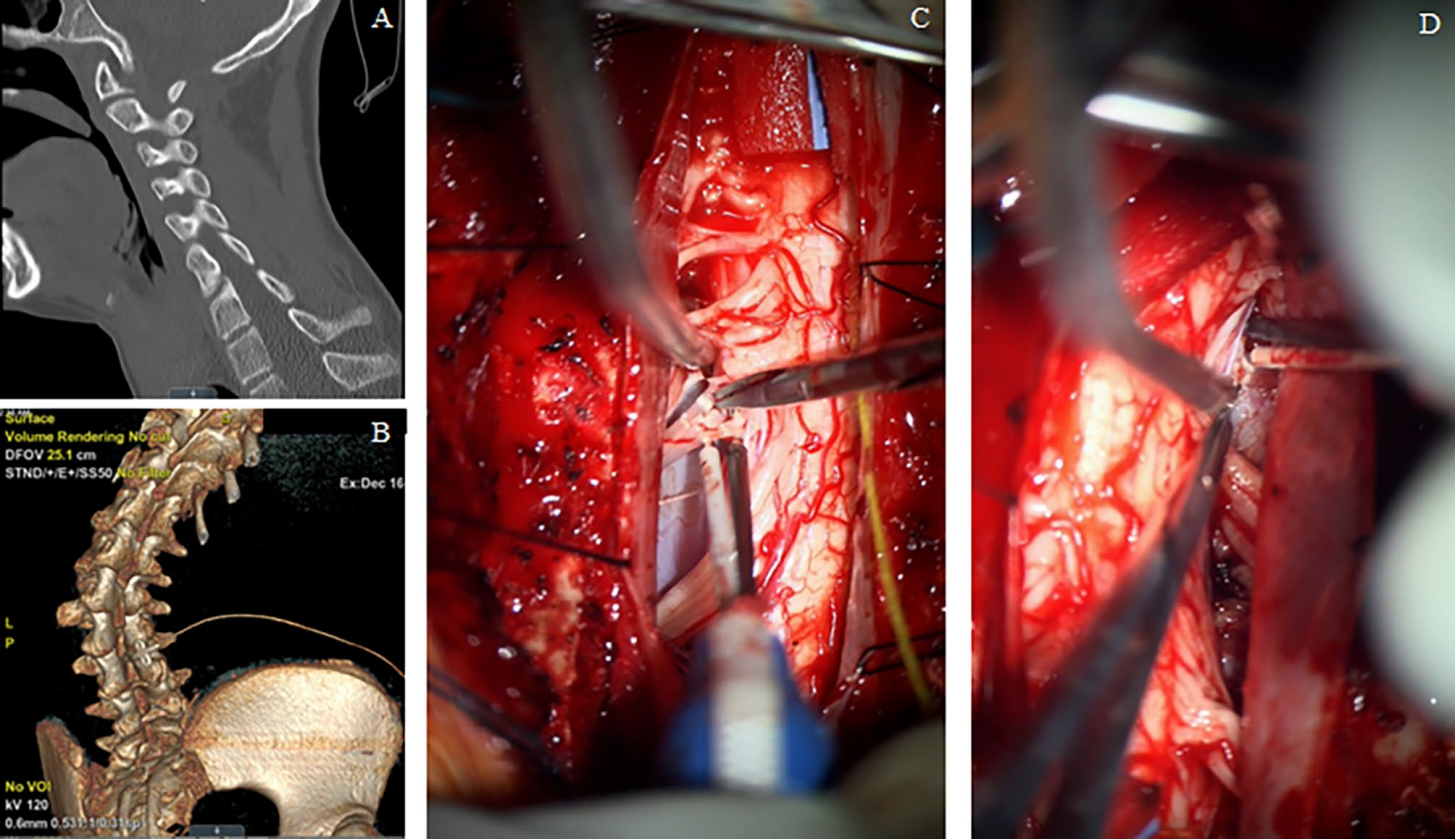
**A** Lower cervical midsagittal CT demonstrates cervical scoliosis; **B** 3-D reconstructed lumbosacral CT demonstrates severe rotatory scoliosis and ITB spinal segment **C** left dorsal and ventral roots easily identified; **D** spinal cord rotation caused by scoliosis makes the right dorsal and ventral nerve roots both difficult to access

A 14-year-old boy presents with mixed quadriplegic spastic, dystonic cerebral palsy secondary to schizencephaly and epilepsy. Despite management with enteral baclofen and valium, botulinum toxin therapy, and ITB his PMD caused progressive hypertonia, musculoskeletal dysfunction, and contractures. His exam was notable for GMFCS 5 functional status, tracks the examiner and communicates by eye gaze device. A multi-disciplinary team determined he had about 30% dystonia and 70% spasticity throughout his extremities with facial and axial dystonia. The muscle groups in his bilateral upper and lower extremities were all rated as modified Ashworth scale (mAS) 4 with fixed contractures across the wrists, knees, and ankles and intertriginous elbow skin breakdown. We performed a single event cervicothoracic and lumbosacral ventral–dorsal rhizotomy as previously described, with Fig. 2 showing some surgical details from the cervicothoracic rhizotomy. His postoperative evaluation at six months was mAS 1–2 throughout his extremities without pain or deformity. The patient and family were delighted.

### Deep brain stimulation for pediatric movement disorders

Academic interest in pediatric DBS has expanded exponentially since the first FDA approval. A Pubmed search for the key words “DBS” and “pediatric” yields 3 results for the year 2003. This number increases to 110 results for 2022 (the last complete year prior to this review) [57–60]. DBS has been performed in several movement disorders in the pediatric population. It has also been used in the treatment of epilepsy [61], depression, obsessive compulsive disorder, eating disorders, and other disease of the nervous system [62]. No large prospective randomized studies investigate the effectiveness of DBS in pediatric movement disorders and most data is extracted from case reports and series. We summarize the data regarding DBS outcomes in primary, secondary, and idiopathic dystonia, tic disorders, choreiform disorders, and tremor.

### Primary dystonia (DYT-1, DYT variants, and other genetic dystonias)

Primary dystonia is the most studied disease treated with pediatric brain stimulation [63]. Similar to pallidotomy, DBS for primary dystonia has the largest treatment effect and response rates in the literature for PMDs. There are over 20 primary dystonia genes named with the Human Genome Organization (HUGO) convention of *DYT* nomenclature [10, 39]. The most common and well-studied of these is DYT1 (TOR1A) [13, 63]. Other genetic causes of movement disorders (e.g., GNAO1) have also been treated with deep brain stimulation with good anecdotal results [64]. 

In the largest meta-analysis to address primary dystonias (analyzing data of 111 patients), 90% of patients showed some degree of improvement after bilateral GPi placement, and 80% of patients showed at least 20% improvement on of the BFMDRS. The median change at last follow-up was 76% improvement on the BFMDRS motor and 70% on the disability score (DFMDRS-D) [13]. 

There is some degree of variability within the group of primary genetic dystonias. DYT1 and DTY6 appear to be best characterized and show robust results. Case reports suggest that there is possibly more variation or a lesser response in other primary dystonias [65]. 

Primary dystonia with degeneration or structural lesions also shows less robust response to DBS treatment. In a meta-analysis of 50 patients, a median improvement of 27% was noted in the DFMDRS motor score and 0% in the disability score [13]. The most common etiology of this group was *pantothenate kinase–associated neurodegeneration (PKAN).* This disease is characterized by progressive iron accumulation within the brain, particularly in the globus pallidus (GP) and pars reticularis of the substantia nigra (SNr). One meta-analysis (73 patients) has suggested the bilateral GPi stimulation may benefit this patient population [66]. As measured by the BFMDRS motor scale, 70% of patients showed at least a 10% reduction in symptoms, and a third for patients shows a 50% or great reduction of symptoms.

### Secondary dystonia

Secondary (or acquired) dystonias occur as the result of another primary disease process. This has been most extensively studied in cerebral palsy (CP) but DBS has also been used as a treatment of dystonia following trauma, stoke, or other brain injuries [67, 68]. CP patients undergoing DBS have shown improvement in their dystonia; however, the magnitude is less than that see in primary dystonia. Patients experience on average at 10–20% improvement in BFMDRS motor scores with a larger proportion of patients experiencing no improvement [13, 69]. 

### Idiopathic dystonia

Idiopathic dystonia occurs in patients where no other identifiable cause of the dystonia has been isolated. It has been proposed that much of this population is composed of genetic dystonias for which a mutation has not yet been characterized. DBS treatment in this group has shown better effectiveness than in secondary dystonia but showed smaller results when compared to primary dystonia. In a meta-analysis (70 children and youths), patients experienced an average improvement of BFMDR motor scores of 50% and disability scores of 39%. Eighty percent of the patients show clinically significant improvement [13]. 

### Tics

A tic is a sudden, rapid, recurrent, non-rhythmic movement or vocalization. Tourette syndrome (TS) is a childhood-onset neuropsychiatric disorder characterized by multiple motor and vocal tics that last at least a year in duration. By definition, onset of the disorder occurs before the age of 18 years. Through the natural history of this disease, many patients will experience improvement or resolution of their symptoms in their 20 s or 30 s [62]. 

Because these symptoms may improve with age, the 2011 European guidelines proposed that surgical interventions should be delayed. Similar positions in Canadian and American guidelines recommend against the use of DBS in children [62, 70]. 

DBS has been used successfully in children with TS young as 12 years of age. No controlled or open trials have been conducted and most evidence comes from small case series. In the largest meta-analysis (58 childrens and youths with an average age 17.9 years), electrodes were placed in the GPi in 33 patients and the centromedian nucleus (CM) of the thalamus in 24 patients. Patients showed on average a 58% improvement in symptoms as measured with Yale global tic severity scale (YGTSS) with a mean follow-up of 34 months. Nearly all children (96.6%) demonstrated some improvement in tic symptoms, with 91.4% and 64.0% showing greater than 20% and 50% improvement, respectively [70]. 

### Choreiform disorders

Chorea from HD does appear to respond to bilateral GPi stimulation in small series of the adult population. In one long-term prospective open-label trial of seven patients, each patient showed improvement after placement of DBS with a mean improvement on the unified Huntington’s disease rating scale (UHDRS) chorea sub-score of 58% at 3 years of follow-up [71]. System reviews however suggest caution about to overall level of improvement in HD [72, 73]. In all analyzed publications, they found a statistically significant improvement of UHDRS chorea sub-score by an average of 40–60% after DBS implantation. However, DBS did not improve functional capacity of HD patients and no systematic assessment concerning the effect of DBS in HD on behavior, cognition or speech was found. Results in the total motor score were poor. While statistically significant improvement was seen at 6 months, these were lost on long-term follow-up. Additionally, these findings may not generalize well to pediatric patients with choreiform disorders. Symptoms of HD tend to develop after the age of 20, and therefore show limited symptoms in the pediatric population. Case reports do suggest some degree of improvement in chorea symptoms pediatric cases of chorea [64, 69]. 

### Tremor

Ventralis intermedius nucleus (VIM) is a common thalamic target for adults suffering from essential tremor. There is strong evidence to support DBS as a treatment for essential tremor in the adult population [74]. However, evidence for this target in the pediatric population exists only at the level of case report data [75]. 

### Example case

We present of case of childhood-onset primary dystonia in a young woman who later underwent bilateral DBS at GPi with good success. Through infancy and preschool ages, she had normal development and was neurotypical with normal gait and age-appropriate self-care. She began to develop clumsiness during kindergarten and experienced frank regression of milestones during the first grade. The dystonia continued to progress, becoming generalized with prominent axial and speech difficulties. This led to the loss of the ability to ambulate and necessitated eventual g-tube placement for nutrition. At age 14, she underwent placement of an intrathecal baclofen pump with limited improvement of her symptoms. At age 17 through participation with the NIH undiagnosed diseases program, she was diagnosed with KMT2B (DYT28) primary genetic dystonia. She was managed medically with oral baclofen, clonazepam, diazepam, and tizanidine in addition to her intrathecal baclofen. Despite maximal medical management, the severity of her dystonia continued to progress resulting in increasing pain and hospitalizations for acute exacerbations of dystonia.

At 22 years of age, she presented for evaluation of deep brain stimulation. She underwent bilateral placement of GPi leads and generators (bilateral Medtronic 3387 leads and Activa generators). The patient was sedated and intubated in the operating room. A Leksell frame (Elektra. Stockholm, Sweden) was affixed to the head. An MRI scan was them performed which included 1-mm (3D) axial slices of T1 with and without contrast, T2, FGATIR, and Turbo IR. The images where then pushed to the Medtronic (Dublin, Ireland) S8 StealthStation. The target location in the bilateral globus pallidus was then determined by a previously published technique [76]. In the anterior commissure (AC)-posterior commissure (PC) plane, the GPi was visualized. The target point was positioned one third of the distance from the posterior–lateral to the anterior–medial point of the GPi and 3 mm anterior to the posterior limb of the internal capsule. An entry point on the skull was selected near the coronal suture which avoided the cortical vessels seen on the post-contrast T1 MRI as well as the lateral ventricles. The *x*, *y*, *z*, arc, and ring coordinates were then obtained from the StealthStation and confirmed by manual calculation based on the measurements from the Leksell frame in MRI using a PACS viewer.

The left lead was placed first after sterilely draping and applying the Leksell ring system to the sedated patient’s head frame. The coordinates were carefully set on the frame and ring system. A semicircular galea incision was performed. The skull was open with a twist drill and the dura opened sharply. A drive headstage (Alpha Omega. Nazareth, Israel) was then affixed to the arc and a microelectrode needle was driven toward the targe while neuronal firing was record by a separate electrophysiologist. The distal position of the lead was determined by the loss of GPi pattern firing. Serial flashlight stimulation was applied to the eyes to exclude inadvertent entry into the optic tracts. A DBS lead was then passed to the target and secured against the skull with a two titanium plates. Its location was confirmed by fluoroscopy. The impedance was checked to exclude damage to the lead and test stimulation was performed through the lead to exclude gross motor side effect from unintended stimulation of the cortical spinal tract. The distal end of the lead was then curled beneath the galea and the process repeated on the opposite side. Final lead positions were confirmed on postoperative CT. Figure 2 demonstrates some of these surgical details. Two weeks following lead placement, the patient underwent generator placement through an infra-clavicular incision.

She experienced a very strong response. She continued to require a g-tube for adequate nutrition but was able to return to taking food my mouth for enjoyment. She regained her ability to ambulate and was able to discontinue her oral medications. Her baclofen pump was slowly weaned to off and removed three years after the DBS system was implanted.

## Discussion

The neurosurgical management of hypertonia is consensus driven and without guidelines; outside of selective dorsal rhizotomy (SDR), there is limited evidence suggesting that early intervention prevents orthopedic deformity, limits the need for future surgery, reduces polypharmacy, or has a mortality advantage [5]. Even so, level III evidence supports neurosurgical management of dystonia in children for tone reduction [27]. 

The reviewed literature suggests that most publications including children for rhizotomy, thalamotomy, and DBS are for indications involving dystonia. In primary dystonia defined by DYT-1 TOR1A gene mutation, both pallidotomy and GPi DBS are highly effective for tone reduction compared to medical management [34, 37, 77]. The available evidence disallows procedural superiority in primary dystonia. Patient-specific factors including very young age at onset, poor functional ability, family declines, or surgical risk factors (e.g., tracheostomy, vagus nerve stimulator, severe torticollis) may influence away from DBS and hardware implantation and towards pallidotomy.

Across studies and historically, bilateral pallidotomy has been associated with complications including bulbar dysfunction, dysphagia, hypophonia, homonymous hemianopsia, and hemiplegia; DBS has mostly obviated these permanent deficits via mostly reversible neuromodulation [37, 43]. Functional patients who communicate by voice and swallow independently may deem these potential side effects unacceptable and prefer DBS.

**Fig. 3:**
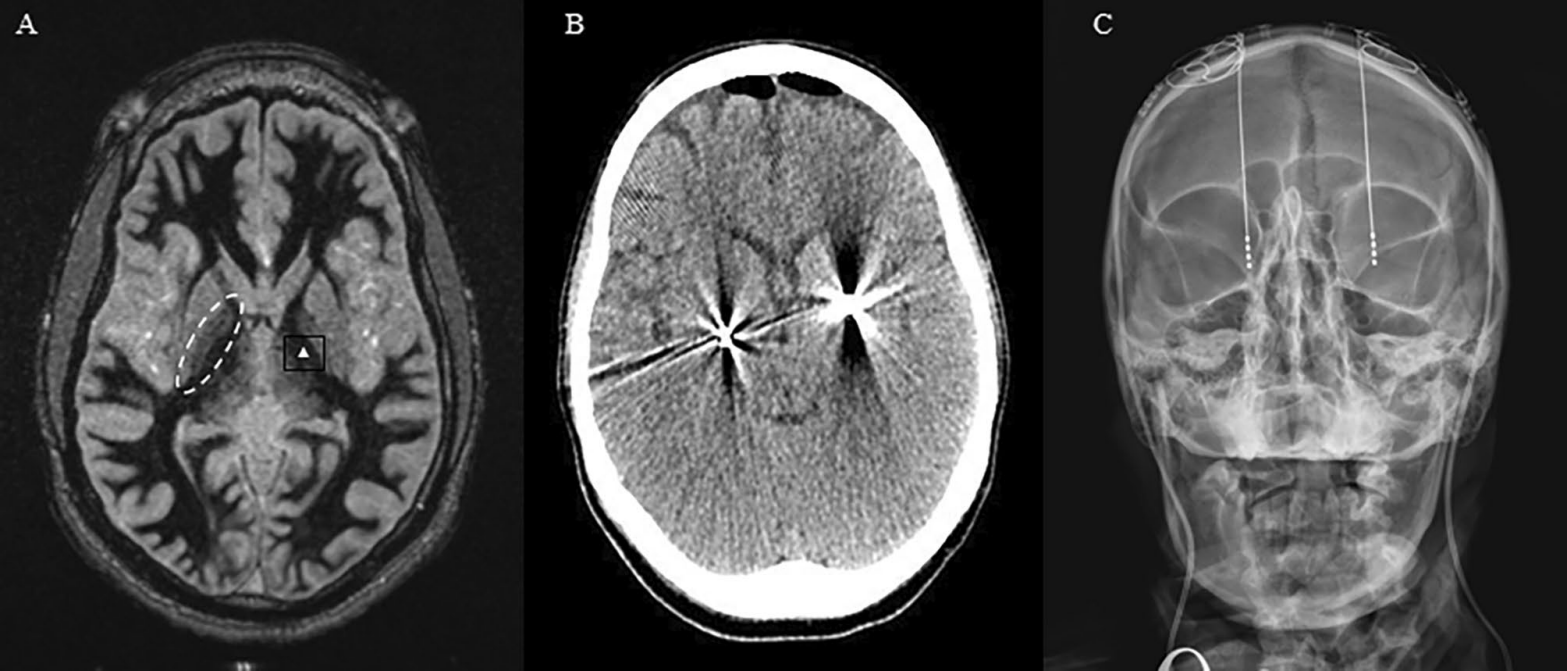
**A** FGATIR MRI sequence used for direct visualization of the GPi (dashed oval). The GPi target is marked with a white triangle. The position was determined using the technique described by Starr and colleagues [76]. Placement of bilateral leads into GPi on postoperative **B** CT scan and **C** x-ray

The morbidity profile of bilateral pallidotomy may be minimized by performing staged pallidotomy [5, 23, 37]. Historically, pallidotomy has been performed at the same DBS target: posteroventral GPi (Fig. 3). It remains an open question regarding central GPi ablation for allowing posteroventral targeting with DBS in failed cases [45]. For secondary dystonia, there seems to be equipoise between pallidotomy and DBS [67, 68]. Both procedures are associated with a paltry 10–25.5% average improvement in dystonia [5, 13, 23, 69].

Secondary dystonic patients do present in status dystonicus, a particularly acute and severe worsening of dystonia defined by dystonic crisis requiring hospitalization and usually requiring significant polypharmacy [78]. Status dystonicus is a severe manifestation of life-threatening dystonia with a mortality rate of 12.5%; neurosurgical management of hypertonia can be life-saving [78]. These critically ill patients have been treated with either pallidotomy or DBS [45, 79]. A single-center study showed equipoise between pallidotomy and DBS rates of ending status dystonicus, 83.3% versus 87.5% respectively, but with a 37.5% rate of hardware complication in the DBS group versus 0% in the pallidotomy group [79]. Multiple studies characterize a higher infection and complication rate for dystonia patients over other patient populations who have DBS [80]. Status dystonicus is an end-stage manifestation of dystonia without benefit of one procedure over another; attempting either pallidotomy or DBS may improve early mortality.

There is extremely limited evidence of pallidotomy in children for movement disorders which do not include dystonia. Initial experience in cases like HD showed initial short-term improvement in choreiform movements, but this effect diminishes as the condition progresses. There is also limited data for pallidotomy in segmental and focal dystonia in children. Level III evidence suggests some marginal effect for using DBS in some patients with choreiform disorders, rare tics, and tremor.

Rhizotomy for dystonia is useful for appendicular tone reduction [50]. Patients who qualify for cervical and/or lumbosacral ventral–dorsal rhizotomy are those who have severe tone which causes pain and limb dysfunction. Ventral–dorsal rhizotomy is a palliative procedure and cannot guarantee any gain of function. Cervical approaches are limited by kyphosis, and in both the cervical and lumbosacral approaches, significant scoliosis or spinal fusion increase the surgical risk significantly. There is very limited modern evidence supporting the long-term benefits, risks, and quality-of-life metrics following ventral–dorsal rhizotomy.

### Limitations

This study is not a comprehensive analysis of movement disorders, but a focused literature review regarding neurosurgical management of non-spastic movement disorders in children. The discussion excludes ITB management of these conditions.

### Future

There remain many questions regarding both pallidotomy and DBS for dystonia. Indications broadly favor “primary” over “secondary” dystonia, and the inexorable expansion of our understanding of the neurogenetic underpinnings will expand the indications of “primary” dystonia patients who improve with neurosurgical treatment. The homogenization of indicated conditions is likely to improve the efficacy of both procedures.

The surgical evolution of pallidotomy may include central GPi targeting, size of the lesion, staged unilateral versus single bilateral procedure, and use of MRI-guided laser interstitial thermal therapy or hi-frequency ultrasonography. Optimal timing of these procedures remains elusive, with some evidence suggesting that early intervention has better outcomes in dystonia. In both cases, pallidotomy and DBS, the identification of biomarkers for objective characterization of outcomes, following long-term clinician measured outcomes, and documenting patient-reported and surrogate reported outcomes will be critical to understanding the impact of neurosurgery.

Surgical complexity is likely to evolve over time. Current NIH studies and other institutional IRBs are applying stereoelectroencephalography to subcortical nuclei with the hope of guiding more effective DBS leads [81]. As our understanding of ablation and neuromodulation matures, salvage therapy might include DBS placed after pallidotomy, or the placement of additional DBS leads into other movement related nuceli [82, 83]. Dual bilateral lesions or dual bilateral DBS may be further investigated.

Finally, neurosurgery for movement disorders may be entirely obviated by gene therapies. A recent systematic review identified 46 published and ongoing studies into gene therapies for PD, HD, and other genetic causes of movement disorders [83]. High volume centers should be encouraged to participate in registries like CHILD-DBS to further this science [59].

## Conclusion

Non-spastic PMDs are common and difficult to treat. The medico-surgical algorithm for treatment includes pallidotomy, DBS, and rhizotomy. Level III evidence suggests that each is effective for tone control. There is very limited data describing the long-term efficacy or complications of ventral–dorsal rhizotomy, and it remains an effective short-term surgical option to denervate the limbs in selected patients. Modern pallidotomy and DBS are both similarly effective in primary dystonia and similarly ineffective in secondary dystonia. There is limited evidence to suggest either for the primary management of choreiform, ataxia, hemiballism, movements.
